# A database on the historical and current occurrences of snakes in Eswatini

**DOI:** 10.1093/database/baaf040

**Published:** 2025-09-24

**Authors:** Ara Monadjem, Richard C Boycott, Thea Litscha-Koen, Adam Kane, Wisdom M Dlamini, Lindelwa Mmema, Katharine L Strutton, Zakhele Hlophe, Sara Padidar

**Affiliations:** Department of Biological Sciences, Kwaluseni Campus, University of Eswatini, Kwaluseni, Eswatini; Mammal Research Institute, Department of Zoology & Entomology, University of Pretoria, Hatfield, 0028, South Africa; Rua do Barreiro No. 7, Freixial do Campo, Castelo Branco, Portugal; Eswatini Antivenom Foundation, Simunye, Eswatini; School of Biology and Environmental Science, University College Dublin, Dublin, Ireland; Department of Geography, Environmental Science and Planning, University of Eswatini, Kwaluseni, Eswatini; Eswatini Antivenom Foundation, Simunye, Eswatini; Eswatini Antivenom Foundation, Simunye, Eswatini; Eswatini Antivenom Foundation, Simunye, Eswatini; Department of Biological Sciences, Kwaluseni Campus, University of Eswatini, Kwaluseni, Eswatini; Eswatini Antivenom Foundation, Simunye, Eswatini; Department of Biochemistry, Genetics and Microbiology, University of Hatfield, 0028, South Africa

## Abstract

Snakes are among the most difficult terrestrial vertebrates to survey, resulting in poor distributional information on most species. This database comprises of 3812 records of 58 species of snakes in 37 genera reported from within the boundaries of Eswatini. The data were compiled from multiple sources including museum specimens, iNaturalist records, literature records, and snake rescue operations. For each specimen reported in the database, we provide the scientific name, latitude and longitude coordinates, and location. Most records also have an associated date. This comprehensive database will be useful to biodiversity experts, conservationists, medical practitioners, researchers, and snake enthusiasts, especially for mapping and modelling snake distributions in the country. To allow easy viewing of the distribution of snakes in the country, we provide an online visualization tool, which should allow a greater number of non-scientists to utilize this database.

## Background and summary

Due to their secretive nature, snakes are typically difficult to survey [1, 2], resulting in poor distributional information for many species, particularly in the tropics [3]. In addition, some snake species are capable of harming or killing humans through envenomation, a risk factor that is perhaps greatly underestimated in parts of the world, including in Africa [4–6]. Spatial analysis is a powerful tool that can be used to map such risks [7, 8], allowing policy-makers to make informed decisions. At the same time, snakes may be far more threatened than previously thought [9]. However, such spatial analyses require maps that accurately represent snake geographic ranges, which are still not available for much of Africa, including Eswatini.

Knowledge on the occurrence of reptiles has a short history in Eswatini (formerly named Swaziland). The first snakes were collected in the 1960s, but systematic surveys did not begin until the late 1980s by one of the authors (R.C.B.), which culminated in two publications listing snake species present in the country [10, 11]. These remain the only published sources of information specifically for snakes occurring in Eswatini, but neither provides locality data. Published distribution records are available for just a handful of species [12–14]. A comprehensive reptile atlas of South Africa, Lesotho, and Eswatini provides maps of geographic ranges for all snakes occurring in this region, but plotted at a coarse resolution of a quarter degree, which roughly translates to 25 km × 25 km [15] that does not allow fine-scale resolution of distributions as needed for a country the size of Eswatini.

Our main aim in this paper is to present accurate information on timestamped snake occurrences in Eswatini, which will allow the plotting of fine-scale distribution maps for each species in the country.

## Methods

### Taxonomic and geographic coverage

**Figure 1.**
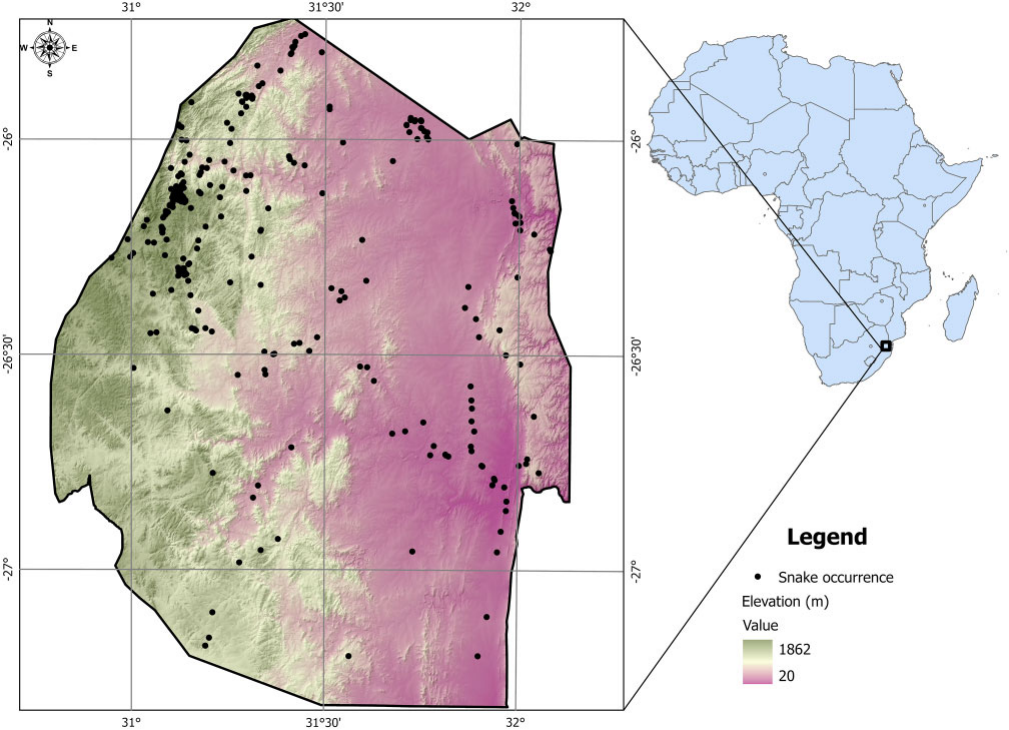
Map of Eswatini with the location of the snakes in this study overlain on a digital elevation map to show the wide range in elevation with highest values in the west and lowest values in the east.

This Eswatini snake dataset includes records of 58 species of snakes in 37 genera (sub-order Serpentes, class Reptilia) collected or observed within the country (Table 1). Eswatini is a small, land-locked country situated between South Africa and Mozambique, covering 17 364 km^2^. The country is topographically heterogeneous resulting in a diverse range of habitats that support over 800 species of vertebrates [16]. Elevation in the eastern Lowveld is 20 m above sea level, and the highest point (Mt. Emlembe) in the north-west is 1862 m above sea level (Fig. 1).

**Table 1.** A checklist of the 58 species of snakes in 37 genera and 12 families occurring in Eswatini

Family	Genus	Species	Conservation status	Medical importance
Atractaspididae	*Amblyodipsas*	*concolor*	Least concern	1
Atractaspididae	*Amblyodipsas*	*polylepis*	Least concern	1
Atractaspididae	*Aparallactus*	*capensis*	Least concern	1
Atractaspididae	*Aparallactus*	*lunulatus*	Least concern	1
Atractaspididae	*Atractaspis*	*bibronii*	Least concern	2
Colubridae	*Crotaphopeltis*	*hotamboeia*	Least concern	1
Colubridae	*Dasypeltis*	*inornata*	Least concern	1
Colubridae	*Dasypeltis*	*scabra*	Least concern	1
Colubridae	*Dipsadoboa*	*aulica*	Least concern	1
Colubridae	*Dispholidus*	*typus*	Least concern	3
Colubridae	*Meizodon*	*semiornatus*	Least concern	1
Colubridae	*Philothamnus*	*hoplogaster*	Least concern	1
Colubridae	*Philothamnus*	*natalensis*	Least concern	1
Colubridae	*Philothamnus*	*occidentalis*	Least concern	1
Colubridae	*Philothamnus*	*semivariegatus*	Least concern	1
Colubridae	*Telescopus*	*semiannulatus*	Least concern	1
Colubridae	*Thelotornis*	*capensis*	Least concern	3
Elapidae	*Aspidelaps*	*scutatus*	Least concern	2
Elapidae	*Dendroaspis*	*polylepis*	Least concern	3
Elapidae	*Elapsoidea*	*boulengeri*	Least concern	2
Elapidae	*Elapsoidea*	*sundevallii*	Least concern	2
Elapidae	*Hemachatus*	*haemachatus*	Least concern	3
Elapidae	*Naja*	*annulifera*	Least concern	3
Elapidae	*Naja*	*mossambica*	Least concern	3
Lamprophiidae	*Alopecion*	*guttatus*	Least concern	1
Lamprophiidae	*Boaedon*	*capensis*	Least concern	1
Lamprophiidae	*Gracililima*	*nyassae*	Least concern	1
Lamprophiidae	*Hemirhagerrhis*	*nototaenia*	Least concern	1
Lamprophiidae	*Homoroselaps*	*dorsalis*	Near threatened	1
Lamprophiidae	*Homoroselaps*	*lacteus*	Least concern	1
Lamprophiidae	*Inyoka*	*swazicus*	Least concern	1
Lamprophiidae	*Lamprophis*	*aurora*	Least concern	1
Lamprophiidae	*Lamprophis*	*fuscus*	Least concern	1
Lamprophiidae	*Limaformosa*	*capensis*	Least concern	1
Lamprophiidae	*Lycodonomorphus*	*inornatus*	Least concern	1
Lamprophiidae	*Lycodonomorphus*	*laevissimus*	Least concern	1
Lamprophiidae	*Lycodonomorphus*	*obscuriventris*	Least concern	1
Lamprophiidae	*Lycodonomorphus*	*rufulus*	Least concern	1
Lamprophiidae	*Lycophidion*	*capense*	Least concern	1
Lamprophiidae	*Lycophidion*	*variegatum*	Least concern	1
Lamprophiidae	*Pseudaspis*	*cana*	Least concern	1
Leptotyphlopidae	*Leptotyphlops*	*scutifrons*	Least concern	1
Leptotyphlopidae	*Leptotyphlops*	*telloi*	Near threatened	1
Leptotyphlopidae	*Myriopholis*	*longicauda*	Least concern	1
Prosymnidae	*Prosymna*	*stuhlmanni*	Least concern	1
Psammophiidae	*Psammophis*	*brevirostris*	Least concern	1
Psammophiidae	*Psammophis*	*crucifer*	Least concern	1
Psammophiidae	*Psammophis*	*mossambicus*	Least concern	1
Psammophiidae	*Psammophis*	*subtaeniatus*	Least concern	1
Psammophiidae	*Psammophylax*	*rhombeatus*	Least concern	1
Pseudoxyrhophiidae	*Duberria*	*lutrix*	Least concern	1
Pythonidae	*Python*	*natalensis*	Least concern	2
Typhlopidae	*Afrotyphlops*	*bibronii*	Least concern	1
Typhlopidae	*Afrotyphlops*	*schlegelii*	Least concern	1
Viperidae	*Bitis*	*arietans*	Least concern	3
Viperidae	*Bitis*	*atropos*	Least concern	2
Viperidae	*Causus*	*defilippii*	Least concern	2
Viperidae	*Causus*	*rhombeatus*	Least concern	2

For each species we also include the number of records in the database, its global regional conservation status [15], and the medical importance of the snake [1 = harmless (no medical intervention normally required), 2 = dangerous (symptomatic medical treatment required), 3 = very dangerous (emergency medical treatment required)].

### Data categories

Each row of the database refers to a different specimen and can be considered a unique record. All records have the following information in separate columns: a unique identifier for each record, the source of the record, the family, genus, and species to which the specimen belongs, latitude and longitude of the location, and the accuracy of the location. Where the specimen was collected and deposited in a museum, we provide the museum accession number. Where available, we provide the date; for some records, only the month and/or year are available, hence we also provide month and year as separate columns. Finally, we indicate whether the record is ‘historic’ for those collected before the year 2000.

The source of each record is either a museum record or an observational record; in the latter case, we record the person or institution responsible for collecting the information pertaining to that record (see ‘Data sources’ section). We follow the taxonomy of Tolley et al. [15] for the family, genus, and species names. The latitude and longitude coordinates are presented in decimal degrees. The location is a description of where the specimen was collected or observed. The accuracy refers to the coordinates and are categorized into 1, 2, and 3. 1 refers to GPS accuracy (<20 m accurate); 2 refers to accuracy of <100 m (as read off Google Earth, for example); and 3 refers to inaccurate (error of up to 1–2 km). We recommend that only records with accuracy of 1 and 2 are used for ecological analyses, whereas accuracy 3 is only recommended for plotting distribution maps and for creating species distribution models.

### Data sources

The Eswatini snake database was compiled by gathering information on all museum specimens (*n* = 739 records). We were only able to locate specimens in the Durban Natural Science Museum, the Ditsong National Museum, and the Eswatini National Museum of Natural History. For these records, we included the accession numbers provided by the museums. Additional records came from the Eswatini Antivenom Foundation (EAF, *n* = 2915 records), iNaturalist records, downloaded via the Global Biodiversity Information Facility [GBIF.org (22 April 2025) GBIF Occurrence Download https://doi.org/10.15468/dl.gg86rq, *n* = 132], and others (*n* = 26 records).

**Figure 2.**
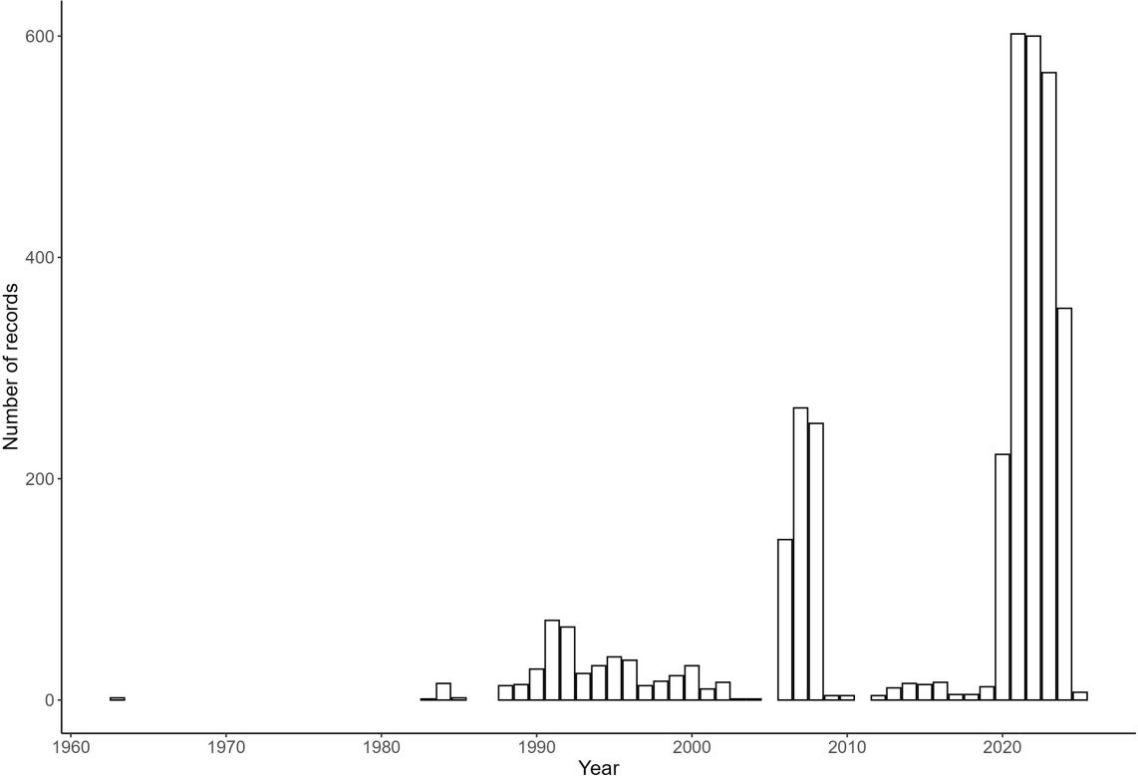
Number of snake records per year in Eswatini since 1963, when the first recorded snake specimen was collected from the country.

The number of snakes recorded per year increased from 1988 when R.C.B. started collecting snakes in the country (Fig. 2). The EAF started collecting data in 2006, but records remained inconsistent until 2021 when over 100 snake rescuers across the country were trained to capture, identify, and photograph snakes as part of EAF’s snakebite prevention strategy. These snake rescue volunteers were trained and equipped by EAF on safe capture, handling, and relocating of venomous and non-venomous snakes. Fifty were trained between October 2021 and February 2022, and a further 50 were trained in February 2024. Snakes were called for capture by the public through a national snake rescue request hotline, and the nearest snake rescuer was deployed to capture and relocate the snake. The snake rescuer reported the location the snake was caught and included a photograph of the snake to EAF for species confirmation. Members of the public also reported sightings of snakes directly to the hotline with a picture and location, without requesting rescue.

### Data records

The Eswatini snake database is available at https://github.com/kanead/snake_database and can be viewed on a map using https://adam-kane.shinyapps.io/Snake_database/.

The metadata for the Eswatini snake database includes the following. Identifier: unique identification number of each record; Source: whether it is a museum specimen or an observation; Museum_number: the accession number of museum specimens; Family: the family of the snake being recorded; Genus: the genus of the snake; Species: the species of the snake; English_name: the English name of the species; Latitude: the latitude in decimal degrees where the snake was captured or observed; Longitude: the longitude in decimal degrees where the snake was captured or observed; Accuracy: the accuracy of the coordinates; Location: the locality where the specimen was first captured/observed; Date: the date of first capture/observation; Month: the month of first capture/observation; Year: the year of first capture/observation; Historic: whether the record was pre-2000 (‘Yes’) or not; Medical_importance: one of three categories (see below).

### Data coverage

**Figure 3.**
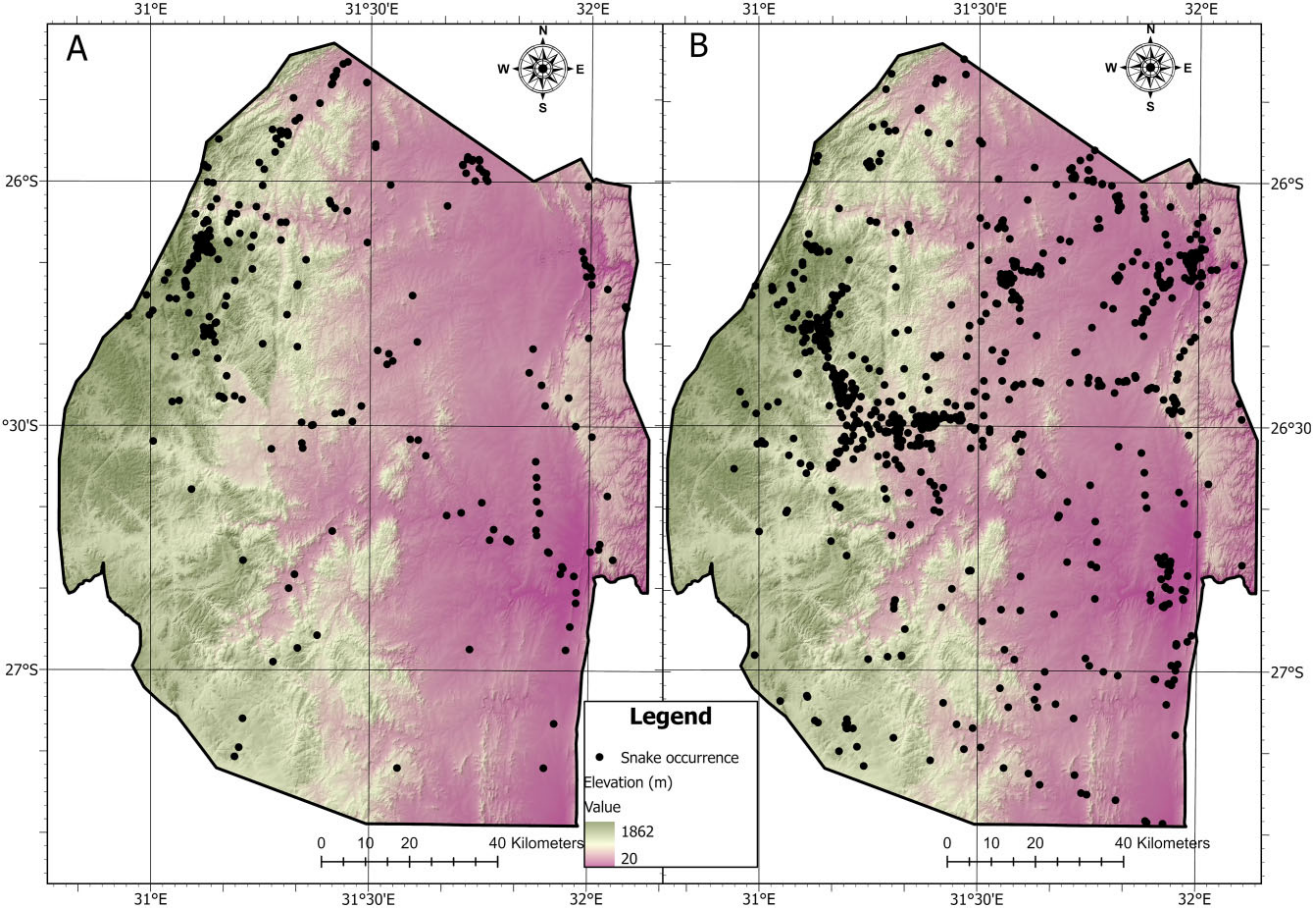
Map showing locations of snake occurrences in Eswatini for (A) historic (pre-2000) and (B) current (post-2000) time periods.

The Eswatini snake database comprises 3812 locality records of all 58 species of snake known to occur in the country (Fig. 3). The snake species, their global IUCN conservation status [17], and their medical importance, taken from the EAF (https://eswatiniantivenom.org/) are presented in Table 1. For human medical importance, we placed each snake into one of three categories: (i) harmless (no medical treatment normally necessary); (ii) dangerous (symptomatic medical treatment required); and (iii) very dangerous (emergency medical treatment essential). A bite from a harmless species is inconsequential for a human and in most cases does not require any medical attention at all (this category also includes some venomous species). A bite from a dangerous species usually requires medical attention but is not life-threatening (this category also includes one non-venomous species). In contrast, a bite from a highly dangerous species should be viewed as life-threatening and treated by a trained medical practitioner acquainted with snake envenomation. It should be noted that repeated exposure to the venom of snakes can cause immunological hypersensitivity leading to anaphylaxis; therefore, snakes classed as 1 or 2 above may occasionally require emergency treatment in a select few individuals.

## Discussion

Despite our best efforts at surveying for snakes across the country, there is an obvious bias towards certain geographical regions. For example, historical records are heavily biased towards Malolotja Nature Reserve, where R.C.B. was the Senior Warden for more than a decade. The more recent records predominantly come from the Manzini–Mbabane corridor area, because this is where the majority of the human population is located, and hence the region where the most calls for snake removal come from. In both time periods, the western and south-western regions of the country received less attention than the north-western and north-eastern regions (where the largest protected areas are situated).

Due to taxonomic problems within the genus *Leptotyphlops*, particularly the *scutifrons*-*conjunctus* complex [15, 18], we have placed all the specimens in this group under the name *Leptotyphlops scutifrons*. Future studies are needed to sort out the taxonomy and systematics of this group, and this will affect the number of species recognized in Eswatini. The recently published ‘A field guide to the snakes of Eswatini’ [19] lists 63 snake species in Eswatini; the five species not occurring in our database are all due to such taxonomic difficulties.

Nearly all the historical records are vouched for by museum specimens. This is not the case for more recent records, the majority of which consist of photographic records instead. It is not always possible to identify a snake by a photograph, and we have only included those records which had identifiable photographs. In addition, prior to 2021 the snake catchers involved in the capture of the snakes did not take GPS points of the localities and frequently did not provide a description of the location. As a result of these two challenges, we have had to exclude more than a thousand records.

### Technical validation

All data were entered manually by either A.M. or S.P. The locations of all the records were checked individually by A.M. to ensure that the localities mapped by the coordinates matched the description of the location.

### Usage notes

The Eswatini snake database is the first accessible and electronic dataset of occurrence records of snakes in Eswatini. The database has been checked for accuracy of location coordinates as well as for the taxonomic identification of each specimen included. We believe that this database will be useful to ecologists, conservationists, data scientists, and health-care practitioners.

Our intention is to update this database regularly and to upload a revised database on an annual basis.

## References

[1] Steen DA . Snakes in the grass: secretive natural histories defy both conventional and progressive statistics. Herpetol Conserv Biol. 2010;5:183–88.

[2] Willson JD, Pittman SE, Beane JC et al. A novel approach for estimating densities of secretive species from road-survey and spatial-movement data. Wildl Res. 2018;45:446–56. 10.1071/WR16175

[3] Marshall BM, Strine CT. Exploring snake occurrence records: spatial biases and marginal gains from accessible social media. PeerJ. 2019;7:e8059. 10.7717/peerj.8059 31871833 PMC6924322

[4] Farooq H, Bero C, Guilengue Y et al. Snakebite incidence in rural sub-Saharan Africa might be severely underestimated. Toxicon. 2022;219:106932. 10.1016/j.toxicon.2022.106932 36181779

[5] Padidar S, Monadjem A, Litschka-Koen T et al. Snakebite epidemiology, outcomes and multi-cluster risk modelling in Eswatini. PLoS Negl Trop Dis. 2023;17:e0011732. 10.1371/journal.pntd.0011732 37948462 PMC10664941

[6] Naik H, Alexander GJ. The incidence of snakebite in South Africa and the challenges associated with lack of reporting. Trans R Soc Trop Med Hyg. 2025;trae109. 10.1093/trstmh/trae109 PMC12212189 39749489

[7] Longbottom J, Shearer FM, Devine M et al. Vulnerability to snakebite envenoming: a global mapping of hotspots. Lancet. 2018;392:673–84. 10.1016/S0140-6736(18)31224-8 30017551 PMC6115328

[8] Pintor AFV, Ray N, Longbottom J et al. Addressing the global snakebite crisis with geo-spatial analyses—recent advances and future direction. Toxicon X. 2021;11:100076. 10.1016/j.toxcx.2021.100076 34401744 PMC8350508

[9] Farooq H, Geldmann J. The fear factor—snakes in Africa might be at an alarming extinction risk. Conserv Lett. 2023;2023:212998.

[10] Boycott RC . An Annotated Checklist of the Amphibians and Reptiles of Swaziland. Mbabane: The Conservation Trust of Swaziland, 1992.;

[11] Boycott RC, Culverwell JB. Swaziland herpetofauna: a preliminary synthesis. J Herpetol Assoc Afr. 1992;40:37–41.

[12] Boycott RC . Notes on the distribution and ecology of *Amblyodipsas concolor* (A. Smith, 1849) in Swaziland (Serpentes: colubridae). Afr J Ecol. 1995;33:419–19. 10.1111/j.1365-2028.1995.tb01050.x

[13] Boycott RC . Further observations on the Natal purple-glossed snake *Amblyodipsas concolor* (Serpentes: Lamprophiidae) in Eswatini with an assessment of its regional status. Afr Herp News. 2018;68:31–36.

[14] Haagner GV, Hurter J. Additional distribution records of the Berg Adder *Bitis atropos* in the south-eastern Transvaal and Swaziland. Koedoe. 1988;31:71–76. 10.4102/koedoe.v31i1.485

[15] Tolley KA, Conradie W, Pietersen DW et al. Conservation Status of the Reptiles of South Africa, Eswatini and Lesotho. Suricata 10. Pretoria: South African National Biodiversity Institute, 2023, 651p.

[16] Monadjem A, Boycott RC, Parker V et al. Threatened Vertebrates of Swaziland. Swaziland Red Data Book: Fishes, Amphibians, Reptiles, Birds and Mammals. Swaziland: Ministry of Tourism, Environment and Communication, 2003, 256p.

[17] IUCN . The IUCN Red List of Threatened Species. Version 2024-1. http://www.iucnredlist.org. 2024.

[18] Adalsteinsson SA, Branch WR, Trape S et al. Molecular phylogeny, classification, and biogeography of snakes of the family Leptotyphlopidae (Reptilia, Squamata). Zootaxa. 2009;2244:1–50. 10.11646/zootaxa.2244.1.1

[19] Wilkey R, Nann S. A Field Guide to the Snakes of Eswatini. Hermit Books, 2024, 294p.

